# Serum calcium level among pregnant women and its association with pre-eclampsia and delivery outcomes: A cross-sectional study from North India

**DOI:** 10.3126/nje.v9i4.23150

**Published:** 2019-12-31

**Authors:** Shashi Kant, Partha Haldar, Anant Gupta, Ayush Lohiya

**Affiliations:** 1 Professor, Centre for Community Medicine, All India Institute of Medical Sciences, Ansari Nagar, New Delhi, India – 110029; 2 Assistant Professor, Centre for Community Medicine, All India Institute of Medical Sciences, Ansari Nagar, New Delhi, India – 110029; 3 Senior Resident, Department of Hospital Administration, All India Institute of Medical Sciences, New Delhi, India; 4 Assistant Professor (Public Health), Super Specialty Cancer Institute & Hospital, Lucknow (Uttar Pradesh), India

**Keywords:** Dietary calcium intake, serum calcium level, pregnancy, hospital

## Abstract

**Background:**

Calcium requirement increases during pregnancy, thereby increasing the chances of developing hypocalcaemia. Hypocalcaemia may be associated with pregnancy-related complications. Therefore, we planned this study to estimate the prevalence of hypocalcaemia among pregnant women attending secondary care hospital, and to study the association between hypocalcaemia and pregnancy outcomes.

**Materials and Methods:**

This study was conducted in a secondary level hospital at Ballabgarh, district Faridabad, Haryana, India. Consecutive pregnant women with gestation period more than 28 weeks were enrolled. Dietary calcium intake was ascertained using 24-hour dietary recall method. Serum calcium estimation was done by Biolis 24i auto analyser. Outcome of pregnancy (preterm delivery, low birth weight (LBW) babies, and neonatal mortality) was assessed telephonically 3 months after the enrolment.

**Results:**

A total of 696 pregnant women were enrolled in the study. Mean (SD) dietary calcium intake and serum calcium level was 796.4 (360.4) mg/day and 9.56 (0.94) mg/dl respectively. Prevalence (95% CI) of hypocalcaemia was 23.9% (20.8 – 27.2%). Serum total calcium level was not associated with dietary calcium intake (p-value = 0.36). Mean serum calcium level was significantly lower in mothers who had LBW babies. Pre-eclampsia, preterm delivery, and neonatal mortality were not associated with serum calcium level.

**Conclusion:**

Serum calcium level may not be related to dietary calcium level. Hence, the current recommendation of calcium supplementation during antenatal period appears to be inconclusive among our study population.

## Introduction

There is an increasing evidence of lower than the recommended daily allowances dietary calcium intake in Indian population [1]. Inadequate dietary intake of calcium may lead to decrease in serum calcium level [2]. Serum calcium level in human body is not only regulated by dietary calcium intake, but, it is also influenced by many other factors like level of parathyroid hormones, vitamin D, and exposure to sunlight [2].

Calcium requirement in non-pregnant state is 600 mg/day which increases to 1,200 mg/day during pregnancy [3]. This increased amount of calcium is required for the growth and development of bones and teeth of fetus. This demand can be met by the increased intake of calcium during pregnancy [3].

Serum calcium level decreases during second and third trimester of pregnancy, primarily due to hemodilution [4]. Some complications of pregnancy may be associated with lower serum calcium level e.g. pre-eclampsia during pregnancy, low birth weight, preterm delivery, and neonatal death [5,6]. Previous studies have reported that pre-eclamptic women have lower serum calcium level as compared to normal pregnant women [7,8,9]. Study done by Gupta et al in North India, however, had concluded that serum calcium level was not associated with pre-eclampsia [10]. The relationship between serum calcium level and pregnancy outcomes is not settled yet. Very few studies in India have reported serum calcium levels among pregnant women [1,10].

Hence, we tried to estimate the prevalence of hypocalcaemia among pregnant women attending antenatal clinic of a secondary level hospital. Secondary objective was to explore the association of serum calcium level with pre-eclampsia, birth weight of the baby, preterm delivery, and neonatal mortality.

## Methodology

### Study design and participants

This hospital based cross-sectional study was conducted in antenatal clinic of a sub-district hospital in Ballabgarh block of district Faridabad, Haryana. Antenatal clinic was held thrice weekly. Average antenatal Out-patient department (OPD) attendance was approximately 120 per day [11]. Study period was from March to May 2015.

### Study tools (Questionnaire, collection of samples, and assessment of vitals)

Eligible pregnant women were administered a self-developed, semi-structured, pre-tested interview schedule. Socio-demographic details, obstetric history, and antenatal history was captured using this interval schedule. Dietary history was ascertained using 24-hour dietary recall method. Dietary calcium intake was estimated using standard nutritive value of Indian foods [12].

Three millilitres of venous blood from ante-cubital fossa was drawn using standard aseptic precautions. Sera was immediately separated by centrifuging at 2000 rpm for 10 minutes, and stored in refrigerator at 4 degree Centigrade. Serum calcium was estimated by enzymatic method using automated spectrophotometer (Biolis 24i manufactured by Carolina Liquid Chemistries Corporation, Winston-Salem, North Carolina, USA). The 24-h dietary recall method was repeated on the second day when the blood report was handed over to the women.

Blood pressure was measured at the first visit using a manual pneumatic sphygmomanometer (Industrial Electronic & Allied Products, Pune, Maharashtra, India), with the pregnant women sitting comfortably as recommended by the British Hypertension Society and the Nurses Hypertension Association. A clean-catch urine sample was tested by dipstick at the first visit to detect albuminuria [13].

### Inclusion criteria

Pregnant women with gestation period of more than 28 weeks were eligible to participate in the study. Eligible women were informed about the purpose of the study and were provided with a Participant Information Sheet (PIS) in local language. Subjects were included after detailed explanation about the study and obtaining written informed consent to participate in this research.

### Exclusion criteria

Women with history of hypertension before 20 weeks of gestation were excluded. Women who were unable to comprehend questions were also excluded from the study. Women with missing values of pregnancy outcomes were excluded from the analysis.

### Sample size calculation

Kumar et al had reported the prevalence of hypocalcaemia among pregnant women as 66% [1]. Therefore, assuming the prevalence of hypocalcaemia to be 66%, with confidence level of 95%, relative precision of 6%, and non-response rate of 20%, the minimum required sample size was estimated to be 715 subjects.

### Outcome and explanatory variable

Pregnancy outcome of all pregnant women was assessed telephonically by one of the investigators of the study (AG). Telephonic assessment was done at least 3 months after the enrolment in the study. We solicited the following information: (i) Place, mode, and date of delivery (ii) Sex, and recorded birth weight of the new-born, and (iii) Survival status of new born at the time of birth (to calculate still birth) and on day 28 (to calculate neonatal mortality). Women were asked to tell about the pregnancy outcomes from the hospital records, if available. Otherwise, they were asked to recall the details of pregnancy outcomes.

### Operational definitions (Explanatory variable & outcome variable)

(i) Preeclampsia was defined as a systolic blood pressure higher than 140 mmHg and a diastolic blood pressure higher than 90 mmHg on two occasions at least 4 h apart after 20 weeks of pregnancy in a woman with previously normal blood pressure, along with proteinuria [defined as ≥1+ on a clean-catch dipstick in the absence of urinary infection] [14]. (ii) Recommended daily allowance (RDA) of calcium for non-pregnant and pregnant women was considered as 600 mg/day and 1,200 mg/day respectively [3]. (iii) Hypocalcaemia was defined as serum calcium level less than 9 mg/dl. (iv) Preterm delivery: Delivery that occurred before 37 weeks of gestation. (v) Low birth weight babies: New-borns with birth weight less than 2,500 grams.

### Quality control

The laboratory technician was well trained and was supervised by a senior staff member of the laboratory. One of the investigators (AG) had received training from Human Nutrition department of AIIMS, New Delhi for 4 days in estimation of dietary calcium intake using 24-hour dietary recall method.

### Ethical committee approval

The study was approved from ethical point of view by Institute Ethics Committee of All India Institute of Medical Sciences, New Delhi. Women who were hypocalcaemic were provided calcium supplements. Women with complications associated with pregnancy were refereed to specialists for further management.

### Data management and statistical analysis:

Data were entered in EpiInfo 7.0.8.3. Data analysis was done using Statistical Package for Social Sciences (IBM Corp. Release 2011. IBM SPSS Statistics for Windows, Version 20.0. Armonk, NY: IBM Corp). Variables were described as proportions or mean (SD), as applicable. To assess the mean difference in serum calcium levels between two groups, t-test was used. P-value of <0.05 was considered as statistically significant.

## Results

### Socio-demographic profile of pregnant women

A total of 696 pregnant women were enrolled in the study. The mean age (SD) of the pregnant women was 23.8 (3.3) years ranged from 18 to 37 years. Almost three-fourth of women were in the age group 18-25 years. More than 90% of women were homemaker. Illiterate women constituted 15.4% of total women (Table 1).

### Obstetric profile of pregnant women

Around one-third of the women were having primigravida, and 15.3% were grand-multigravida. Among multi-gravida women, 45.0% had history of one or more abortions. Proportion of pregnant women with high risk factors was as follows: 13.1% had pre-eclampsia, 18.2% were short statured(<145 cm).The mean haemoglobin level (SD) was 9.60 gm/dl (1.13). Higher proportion of pregnant women had anaemia (90.7%) and the frequency of severe anaemic women was found to be 2.6% (Table 1).

### Dietary calcium intake and serum calcium level among pregnant women

The mean dietary calcium intake (SD) was 796.4 (360.4) mg/day. Only 18% women had dietary calcium intake more than the recommended amount of 1200 mg/day. Mean (SD) serum calcium level among pregnant women was 9.56 (0.94) mg/dl. The prevalence (95% CI) of hypocalcaemia (serum calcium <9 mg/dl) was 23.9% (20.8 – 27.2%) (Table 2). There was no difference in mean serum calcium level of those who had adequate dietary calcium intake as compared to those who had inadequate dietary calcium intake (p-value – 0.13) (Table 3). Serum calcium level was not correlated with dietary calcium intake (p-value – 0.36, r – 0.04).

### Pregnancy outcome

Data on the pregnancy outcome was available for 668 (96.0%) of the total 696 women. Almost 7% pregnant women had delivered at home. Approximately one-fifth of pregnant women delivered by caesarean section. Eighteen percent of women had preterm deliveries. The proportion of low birth weight new-born was 18.4%. Males baby constituted 56.6% of all births. There were 659 live births and nine still births. Out of 659 live new-born, 14 died within 28 days of birth so the neonatal mortality rate was 21.2 per 1000 live births (Table 4).

### Distribution of serum calcium level

Mean serum calcium level was lower among pregnant women who delivered a low birth weight new-born as compared to women who had normal weight new-born. This difference was statistically significant. None of the other pregnancy outcomes had statistically significant difference in mean serum calcium level or dietary calcium intake (Table 5).

When hypocalcaemic group was compared with normocalcaemic group, the prevalence of preeclampsia was same in both the groups (12.6%). The prevalence of preterm birth was higher in hypocalcaemic group (21.93%) as compared to normocalcaemic group (17.0%) but the difference was not significant (p-value – 0.23). The prevalence of low birth weight was also higher in hypocalcaemic group (24.78%) as compared to the normocalcaemic group (17.0%) but there was no significant statistical difference between the two groups (p-value – 0.06). Similarly the difference in the prevalence of neonatal mortality between hypocalcaemic and normocalcaemic group was non-significant (p-value – 0.25) (Table 6).

## Discussion

### Summary of findings

This study provided information on dietary calcium intake, mean serum calcium level, and the prevalence of hypocalcaemia among pregnant women attending antenatal clinic of a secondary level hospital. Only 18% of pregnant women had recommended daily dietary calcium intake. Prevalence of hypocalcaemia was 23.9%. We also explored the association of serum calcium levels, and dietary calcium intake with pre-eclampsia and other pregnancy outcomes. Mean serum calcium level among pregnant women who delivered LBW baby was significantly lower than those who delivered a baby with birth weight 2500 grams or more.

### Dietary calcium intake in study population

Mean (SD) dietary calcium intake was 796.4 (360.4) mg/day. It was less than the recommended daily allowance (RDA) of 1,200 mg/day for pregnant women. Studies done outside India have also reported similar results [5,15,16,17]. The mean daily dietary calcium intake reported by Kumar was fairly low (324.4 (199.5) mg/day). Though the mean dietary calcium intake was lower than RDA, the mean serum calcium was within normal range for 75% of the pregnant women. Kumar had estimated the dietary calcium intake using average of 3 day and 24-hour dietary recall [1] whereas we had assessed dietary intake using two day 24-dietary recall. Two day dietary recall is considered adequate to assess average daily dietary intake. The standard deviation around the mean value was large in both the studies. Therefore, our findings were within the range of daily calcium intake reported by Kumar et al. Earlier we had conducted similar study in adjacent area and had reported that mean dietary calcium intake as 858.4 mg/day. Our findings are consistent with the previously reported study [10]. Moreover, the standard deviation was wide and the lower bound of the 95% CI in our study was well within the range reported by Kumar [1].

### Serum calcium level in pregnant women

The observed mean (SD) serum calcium level was 9.56 (0.94) mg/dl which was within the normal range for pregnant women (9-11 mg/dl). Kumar et al from New Delhi reported the mean serum calcium among antenatal women as 8.1mg/dl, which was lower than the normal range [1]. Kumar et al did this study in a tertiary care hospital in Central Delhi. It is likely that most of the patients were resident of urban area. In our study, women from rural area were also included. Residents of rural areas generally have higher exposure to sun [18]which might increase vitamin D formation in the body. Higher level of vitamin D facilitates increase the serum calcium level. Therefore, one possible explanation for difference in mean serum calcium level could be area of residence. Though this proposition requires further exploration [10]. Tertiary care hospitals are often referral centres which might attract pregnant women with other pre-existing co-morbidities that may impact serum calcium level. So, another possible reason for lower mean serum calcium level Kumar’s study could be differences in morbidity status of participants [1].

Though the mean dietary calcium intake was lower than RDA, the mean serum calcium was within normal range. This could be because the serum calcium level is not directly correlated with dietary calcium intake [1,10,19]. Dietary calcium is one of the factors involved in maintaining serum calcium level. It is also affected by other factors such as levels of parathyroid hormone, 1, 25-dihydroxy vitamin D, among others [2].

### Association of delivery outcomes with serum calcium levels in study population

We also explored the association of serum calcium levels and daily dietary calcium intake with pre-eclampsia and adverse birth outcomes (preterm birth, low birth weight, neonatal mortality). Low serum calcium level has been implicated as risk factor for pre-eclampsia in many previous studies [7,8,9,20,21]. However, in this study, we did not find hypocalcaemia to be associated with pre-eclampsia. Reasons for it could be: (i) Sample size for this study was calculated to estimate the prevalence of hypocalcaemia. Thus, the study might not be sufficiently powered to detect the association even if truly existed. (ii) We assessed the pregnant women for pre-eclampsia only on one occasion. It is likely that some women might have developed it after our assessment. (iii) Pre-eclampsia is diagnosed after 20 weeks of gestation. Some other studies have also reported that there is no effect of calcium supplementation on pre-eclampsia [22,23,24]. An et al did meta-analysis and concluded that there is no effect of calcium supplementation on pre-eclampsia [24]. Hence, it is not surprising that we did not find any association of dietary calcium intake or hypocalcaemia with pre-eclampsia.

We found that mothers of low birth weight babies had mean serum calcium level that was lower than mothers of babies that weighed 2500 grams or more. Some studies report that low maternal serum calcium level was associated with higher incidence of low birth weight of the baby, pre-term delivery, and neonatal mortality [5,6]. We also found that low serum calcium level was significantly associated with low birth weight babies. However, we did not find any statistically significant association for other birth outcomes. Previous meta-analyses have also reported that pre-eclampsia, pre-term delivery, low birth weight, and neonatal death were not affected by calcium supplementation [24].

This study was done with adequate sample size. All the samples were tested in one laboratory which had vast experience of doing this test. Since this study was done in a hospital setting, pregnant women with poor access to health services could have been missed. Since pregnant women were assessed for pre-eclampsia only once after 28 weeks, we might have missed some cases of pre-eclampsia leading to under-estimation of the disease prevalence.

## Conclusion

We conclude that the serum calcium level was adequate among pregnant women (only 24% had hypocalcaemia) attending antenatal clinic of secondary care hospital though their daily dietary calcium intake was inadequate. Low birth weight was significantly associated with low serum calcium level.

### Limitation of the study:

We have assumed that the missing values were randomly distributed and there was no systematic bias. If this assumption was incorrect, then we might have missed an association even if it truly existed. Seventy-three percent (73%) of all deliveries had taken place in hospital and therefore the birth weight of the new born was available for majority of the new born. Birth weight of most of the home delivered new born was not available, hence they were excluded from analysis. We do not know if women who delivered at home had systematically different dietary calcium intake as compared to those women who had institutional delivery. However, since we have already shown that there was no correlation between dietary calcium intake and serum calcium level, exclusion of home delivered new born would not affect the findings of the study. Delivery at home indicates poor access to health care facilities. Socio-economically disadvantaged women have poor access to health care facility and therefore more likely to deliver at home. The birth weight of the new born is influenced by the maternal nutrition which is poor in low socio-economic group. Hence a larger proportion of LBW new born of women who delivered at home may have been missed. Serum calcium level has not been shown to be associated with low socio-economic status. Therefore, though we excluded the new born delivered at home due to non-availability of birth weight, the main finding is likely to be valid.

### Future scope of the study:

In this study, it was found that the serum calcium was associated with birth weight of the new born. However, the dietary calcium intake was not associated with birth weight and other neonatal outcomes. There is a need to explore the effect of dietary calcium on serum calcium level and birth outcomes. This information can then be used to understand the role of calcium supplementation during pregnancy.

### What is already known on this topic?

Previous studies have discussed the effect of serum calcium on pre-eclampsia and pregnancy outcomes. Some studies have demonstrated the association of serum calcium with pregnancy outcomes. On the other hand, some studies have not found any association.

### What this study adds:

This study describes the relationship of serum calcium with pre-eclampsia and other pregnancy outcomes. Serum calcium was significantly less in mothers who delivered a low birth weight new born as compared to mothers who delivered a new born weighing 2500 grams or more. The study also demonstrates that the serum calcium is not associated with dietary calcium intake. Pre-eclampsia and pregnancy outcomes were also not found to be related to dietary calcium intake.

## Figures and Tables

**Table 1:** Distribution of pregnant women by socio-demographic and clinical variables (N-696)

	Categories	Number (Percentage)
Age group (in years)	18 – 21	190 (27.3)
	22 – 25	343 (49.3)
	26 – 29	107 (15.4)
	>=30	56 (8.0)
Occupational status	Home maker	664 (95.4)
	Others	32 (4.6)
Educational status	Intermediate and above	227 (32.6)
	High school	155 (22.3)
	Middle	114 (16.4)
	Primary	93 (13.4)
	Illiterate	107 (15.4)
Gravidarum	1^	245 (35.2)
	2	207 (29.7)
	3	138 (19.8)
	>=4	106 (15.3)
No. of abortions * (n=451)	0	248 (55.0)
	1	158 (35.0)
	2	33 (7.3)
	>=3	12 (2.7)
Presence of high-risk factors	Pre-eclampsia	91 (13.1)
	Severe anaemia	18 (2.6)
	Height <145 cm	127 (18.2)
	Anaemia Mild anaemia Moderate anaemia Severe anaemia	631 (90.7) 101 (14.5) 512 (73.6) 18 (2.6)

^ Primigravida.

* Primigravida were excluded.

**Table 2:** Distribution of pregnant women by dietary calcium intake and serum calcium level (N=696)

Dietary calcium intake (in mg/day)	Number (Percentage)
<400	112 (16.1)
400-799	248 (35.6)
800-1,199	211 (30.3)
>=1,200	125 (18.0)
**Serum Calcium level (in mg/dl)**	**Number (Percentage)**
<9	166 (23.9)
9-10	365 (52.4)
>10	158 (22.7)
Missing value	7 (1.0)

**Table 3:** Distribution of serum calcium level by daily dietary calcium intake (N = 689*)

Daily dietary calcium intake	Serum calcium level		p value
	<9 mg/dl (N = 119)	>=9 mg/dl (N = 570)	
**<1200 mg/day (N = 566)**	105 (18.6)	461 (81.4)	0.13
**>=1200 mg/day (N = 123)**	14 (11.4)	109 (88.6)	

* Serum calcium level were missing for 7 women.

**Table 4:** Distribution of pregnant women by pregnancy outcome (N-668)

	Variables	Number (Percentage)
Place of delivery*	Home	46 (6.9)
	Government hospital	459 (69.0)
	Private hospital	161 (24.1)
Mode of delivery	LSCS^	124 (18.5)
	Normal vaginal delivery	544 (81.5)
Duration of pregnancy	Pre-term birth	120 (18.0)
	Term/ post-term birth	548 (82.0)
Birth weight of the baby	Low birth weight baby	123 (18.4)
	Normal weight baby	545 (81.6)
Sex of the baby	Male baby	378 (56.6)
	Female baby	290 (43.4)
Outcome of delivery	Still birth	9 (1.3)
	Live birth	659 (98.7)

^ - lower (uterine) segment Caesarean section

* - denominator is 666

**Table 5:** Association between pregnancy outcomes and serum calcium level and dietary calcium intake

Categories		Mean (SD) serum calcium levels (mg/dl)	p value
**Pre-eclampsia**	Yes (N-91)	9.5 (0.9)	0.74
	No (N-598)	9.6 (1.0)	
**Preterm Birth**	Yes (N-118)	9.4 (0.9)	0.13
	No (N-543)	9.6 (1.0)	
**Low birth weight baby**	Yes (N-121)	9.4 (0.9)	0.03*
	No (N-540)	9.6 (0.9)	
**Neonatal mortality**	Yes (N-14)	9.5 (0.8)	0.64
	No (N-638)	9.6 (0.9)	
**Categories**		**Mean (SD) dietary calcium intake (mg/day)**	**p value**
**Pre-eclampsia**	Yes (N-91)	801.7 (357.4)	0.32
	No (N-605)	761.5 (379.5)	
**Preterm Birth**	Yes (N-120)	789.8 (374.3)	0.85
	No (N-548)	796.5 (356.9)	
**Low birth weight baby**	Yes (N-123)	796.9 (364.7)	0.95
	No (N-545)	795.0 (359.0)	
**Neonatal mortality**	Yes (N-14)	777.7 (388.9)	0.50
	No (N-645)	793.6 (360.1)	

**Table 6:** Association between pregnancy outcomes and hypocalcemia

Categories		Serum calcium level		p value
		<9 mg/dl	>=9 mg/dl	
**Pre-eclampsia**	Yes (N – 87)	15 (12.61)	72 (12.63)	1.00
	No (N – 602)	104 (87.39)	498 (87.37)	
**Preterm Birth**	Yes (N – 118)	25 (21.93)	93 (17.0)	0.23
	No (N – 543)	89 (78.07)	454 (83.0)	
**Low birth weight baby**	Yes (N – 121)	28 (24.78)	93 (17.03)	0.06
	No (N – 538)	85 (75.22)	453 (82.97)	
**Neonatal mortality**	Yes (N – 14)	4 (3.57)	10 (1.85)	0.28
	No (N – 638)	108 (96.43)	530 (98.15)	
